# Supplementary material for: Virus-Induced Plant Volatiles Promote Virus Acquisition and Transmission by Insect Vectors
Source: Int J Mol Sci. 2023 Jan 16;24(2):1777. doi: 10.3390/ijms24021777 (PMC9860585; doi:10.3390/ijms24021777)

Virus-induced plant volatiles promote virus acquisition and transmission by insect vectors

Xuefei Chang[1,2], Yating Guo[1], Yijia Ren[1], Yifan Li[1], Fang Wang[2], Gongyin Ye[2]*, Zhaozhi Lu[1]*

[1]Shandong Engineering Research Center for Environment-Friendly Agricultural Pest Management, College of Plant Health and Medicine, Qingdao Agricultural University, Qingdao 266109, China

[2]State Key Laboratory of Rice Biology & Ministry of Agriculture and Rural Affairs Key Laboratory of Molecular Biology of Crop Diseases and Insects, Institute of Insect Sciences, Zhejiang University, Hangzhou 310058, China

***Correspondence:**

Gongyin Ye, email: chu@zju.edu.cn

Zhaozhi Lu, email: zhaozhi@qau.edu.cn

Methods

Population of viruliferous *Nephotettix virescens*

To obtain viruliferous *N. virescens*, non-viruliferous nymphs were confined with RDV-infected Taichung Native1 (TN1) rice seedlings for 72 h and then transferred to virus-free TN1 rice seedlings to infect these plants with the virus. To ensure the insects were viruliferous, nymphs were individually released into separate glass tubes (D = 2.5 cm; H = 25 cm) with one TN1 rice seedling that was given a reference number. Seedlings were replaced every 48 h and those seedlings were transplanted in the greenhouse and grown under natural lighting at a temperature of 25 ± 2 °C so as to maintain a reservoir of the virus; the RDV infection status of these rice plants was determined by RT-PCR. Approximately 20 days later (incubation period of RDV in rice plants), when RDV symptoms became apparent, viruliferous *N. virescens* were selected and maintained on the RDV-infected rice plants in a climate chamber at 26 ± 1 °C, 70 ± 5% relative humidity, under a regime of 14 h:10 h (light: dark).

RDV-infected rice plants

4th or 5th instar viruliferous *N. virescens* nymphs were placed into plastic tubes using a transparent tube (each containing one nymph/seedling) (WT-RDV, *oscas*1-RDV) for 72 h. For consistency, mock-inoculated seedlings (WT, *oscas*1) were infested with non-viruliferous *N. virescens* nymphs as controls. All these seedlings were clean out and transferred into the greenhouse growing as conditions above, and were used for experiments when they were 40 d old.

Figure S1. Heat maps of the walking tracks by non-viruliferous and viruliferous *N. virescens* populations (A, B) exposed to WT and WT-RDV rice plant odors over a 20 min period.

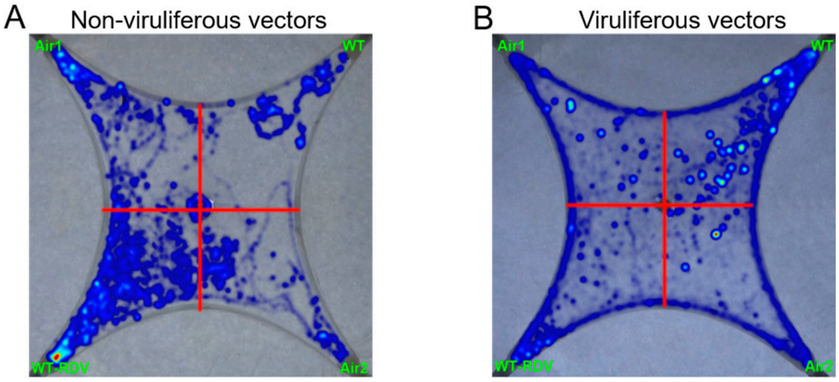

Figure S2. Heat maps of the walking tracks by non-viruliferous (A) and viruliferous *N. virescens* populations (B) in filled with EBC (0.1 µg µL^{-1}), and by the non-viruliferous (C) and viruliferous *N. virescens* populations (D) in filled with 2-heptanol (0.1 µg µL^{-1}), in four-field olfactometer over a 20 min period.

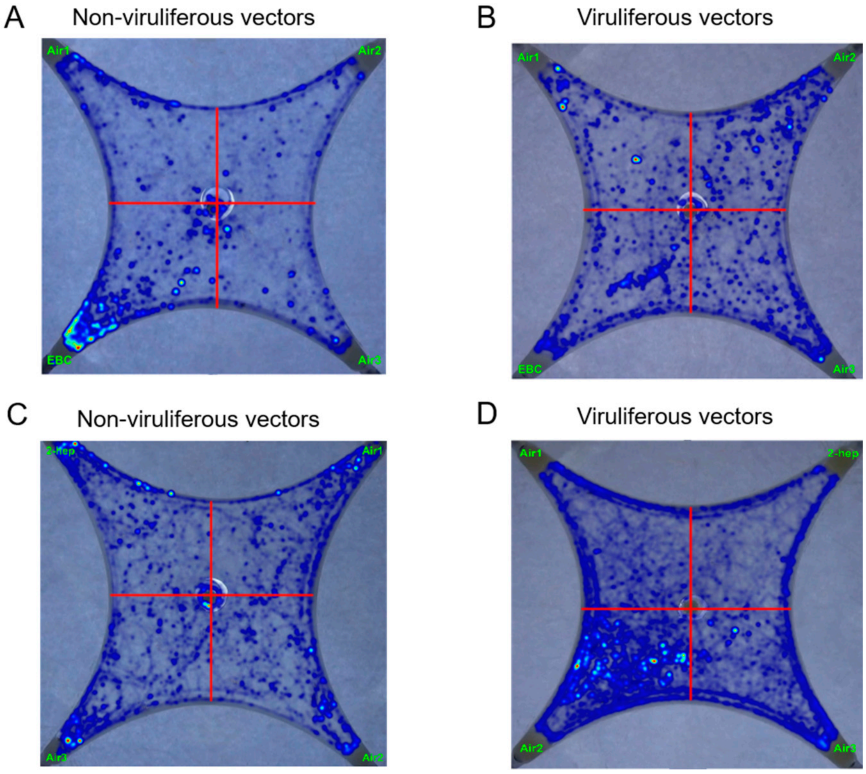

Figure S3. Heat maps of the walking tracks by non-viruliferous *N. virescens* exposed to WT and oscas1 rice plant odors (A), oscas1 and oscas1-RDV rice plant odors (B), WT-RDV and oscas1-RDV rice plant odors (C) over a 20 min period.

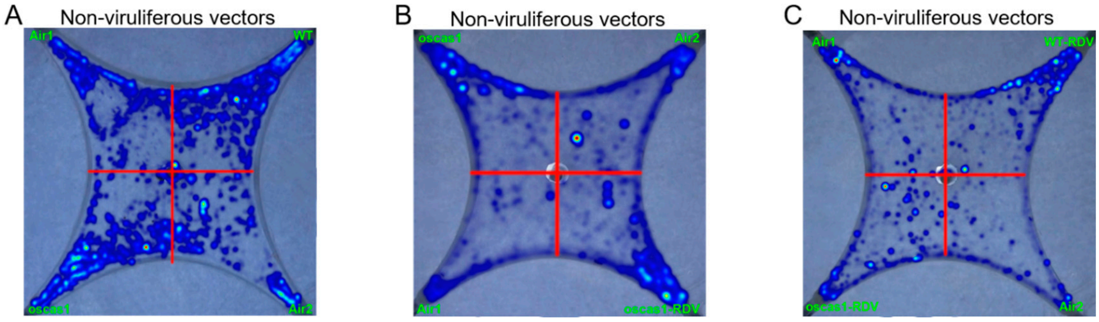

Figure S4. Heat map of the walking tracks by viruliferous *N. virescens* exposed to WT rice plants and WT+2-heptanol rice plants.

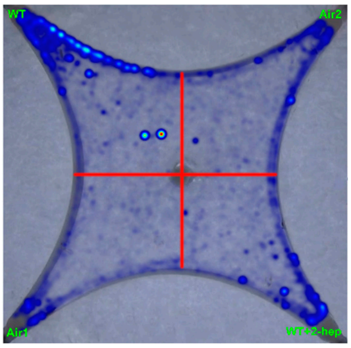

Figure S5. The experimental device of the feeding preference studies.

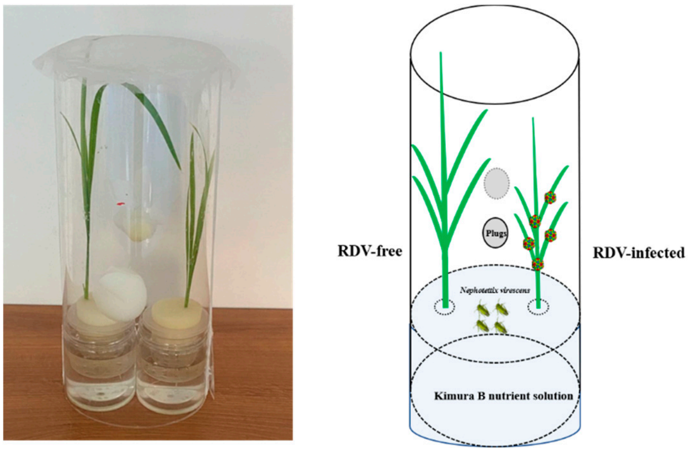

Figure S6. The experimental device of the plant odor preference studies.

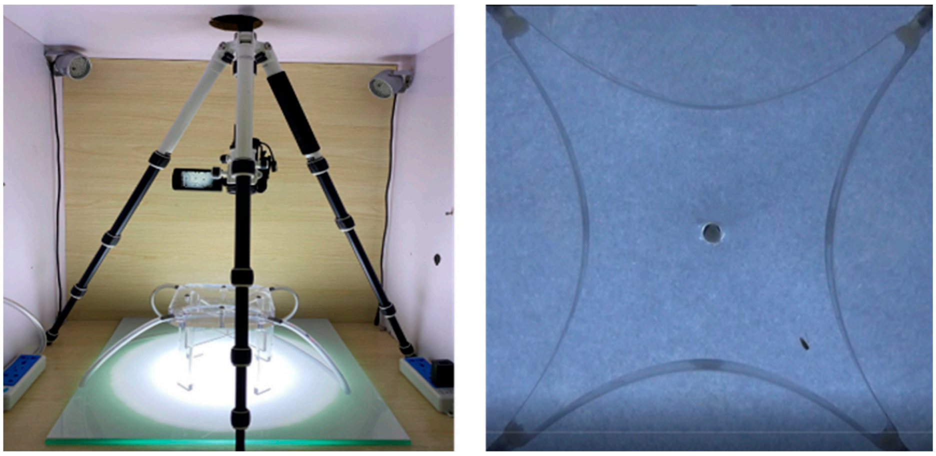

Figure S7. The experimental device of the rate of RDV transmission by viruliferous *N. virescens* between WT plants plus 10 μL pure lanolin paste (WT) and WT plants plus synthetic 2-heptanol (2-hep, 8.17 μg) in 10 μL of lanolin (WT+2-heptanol).

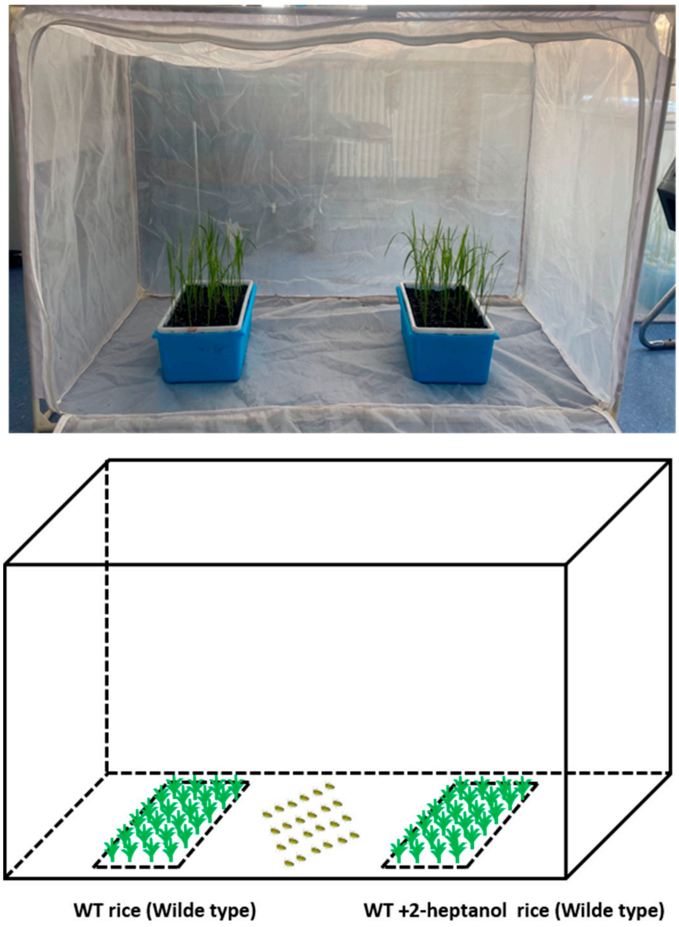

Supplement: Supplementary file 1 [file ijms-24-01777-s001.zip › ijms-2140164-Supplementary information.pdf]
